# Racial re-inscriptions? Examining the potentials and limitations of self-identification variables in German survey research

**DOI:** 10.3389/fsoc.2025.1562478

**Published:** 2025-07-23

**Authors:** Tae Jun Kim

**Affiliations:** German Center for Integration and Migration Research, Berlin, Germany

**Keywords:** quantitative methods, racism, self-identification, survey, racism theories

## Abstract

In recent years, quantitative methods have become a central tool in the fight against racism in Germany, offering empirical evidence to support anti-discrimination efforts. Among these methods, especially self-identification has gained prominence as a way to capture racialized experiences more accurately and to empower marginalized visibilities. By drawing on critical theories of racism, this paper argues that while self-identification may appear to challenge essentialist thinking, it can also risk re-inscribing race by stabilizing fluid identities into fixed categories. These risks are particularly salient in the German context, where official statistics have historically avoided racial categorization, and where recent shifts toward race-sensitive data collection raise new ethical and epistemological questions. By engaging with current methodological debates and highlighting both the contributions and limitations of quantitative approaches, this analysis calls for more reflexive, context-sensitive, and theoretically informed research designs.

## 1 Quantitative methods: a new tool to combat racism?

The year 2020 marked a pivotal moment in Germany's discourse on racism, catalyzed by far-right terrorist attacks in Halle, Hanau and the global resonance of the Black Lives Matter movement following George Floyd's murder. These events pushed racism from the margins of public discourse into the spotlight, prompting its recognition as one of the key societal challenges in contemporary Germany (della Porta et al., 2023; Zajak et al., 2021, 2023). This shift was further institutionalized through the work of the 2020 Cabinet Committee, which formally integrated the term racism into the country's legal framework. These changes not only influenced policy but also led to increased funding for racism monitoring and a broader governmental commitment to address racial inequalities (DeZIM, 2023a).

At the center of this transformation has been the growing reliance on quantitative data. For many civil society and marginalized communities, the adoption of quantitative methods was long overdue, as political responses to social problems in Germany often depend on whether those problems are deemed important enough and supported by empirical data (Supik, 2023). The phrase “Whoever is not counted, does not count” (Aikins et al., 2020, p. 18) has thus gained new significance. Quantitative measures are increasingly viewed as powerful tools in anti-discrimination efforts, providing evidence for racial disparities and helping to shape less polarized and data-driven public debates. Large-scale surveys have become the preferred instruments to capture experiences of racism and compare them across social groups (Supik, 2017), reinforcing the role of numbers as a mechanism for social recognition and policy legitimation.

Yet, the growing prominence of quantitative approaches also raises significant methodological and conceptual questions. Thus, at the heart of this “quantitative turn in racism research” lies a major problem that has yet to be solved: Who is being measured as the subject of racism, and how should that subject be defined? This question is, of course, not new, as it echoes ongoing debates about the fit of migration variables, which have long served as proxies for race in Germany. The category of “migration background,” in particular, has faced increasing criticism for its inability to capture the full spectrum of racialization. It distinguishes individuals based solely on whether they or their parents were born abroad, failing to account for the racial experiences of people who lack a migration background (Ahyoud et al., 2018). For example, a Black person whose parents were both born in Germany does not qualify for having a migration history yet is still subject to racial discrimination. At the same time, not everyone with a migration background is racialized in the same way. “White” migrants from Western Europe or North America, for instance, may not experience racialization at all. These dual limitations highlight why migration-related variables cannot adequately capture the dynamics of race and racism in Germany—particularly as the country's population grows more diverse in the future (Will, 2022).

In response to these limitations, quantitative researchers have begun to explore alternative approaches. One such measure is reflexive ascription, which asks individuals whether they are typically perceived as foreign or non-white in their daily lives (Aikins and Supik, 2018). Simultaneously, self-identification has gained traction as a survey variable that allows respondents to reveal their own racialized positionality. Several high-profile studies in Germany have adopted this approach to monitor the extent of racial discrimination and its consequences for those affected (Aikins et al., 2021; DeZIM, 2023b; European Union Agency for Fundamental Rights, 2023). While these efforts represent significant methodological improvement, scholarly attention has largely focused on technical refinement—on how to improve the accuracy or reliability of such measures—rather than critically reflecting their epistemological implications.

This paper addresses that gap. Specifically, it examines how self-identification—despite its emancipatory promise—may also carry the risk of re-inscribing race as a stable, measurable trait. While self-identification is often seen as a flexible and agency-affirming concept, it can produce essentialist effects when uncritically translated to statistical instruments and categories. This concern is particularly acute in the German context, where racial categories have historically been avoided in official data collection due to the country's National Socialist past. As Germany cautiously begins to reintroduce race-relevant categories through self-identification, it becomes crucial to reflect on the risks such measures might entail. In addition, the adoption of quantitative methods to study racism in Germany has occurred without significant reference to critical theories of racism. These theories, while marginal in public German discourse, have been influential in academic debates since the 1980s (Kalpaka and Räthzel, 1990). As a result, newly employed quantitative measures for race often lack a deeper theoretical discussion that focuses on the very side-effects that concepts such as self-identification can introduce into our academic thinking.

Drawing on critical theories of race and racism, this paper argues that quantitative researchers—wittingly or not—participate in the construction of racial knowledge. Survey instruments do not merely capture social reality; they help produce it. The task, therefore, is not to reject quantitative methods outright, but to interrogate their role in reinforcing or challenging dominant racial logics. By focusing on the case of Germany, this analysis offers a theoretically grounded and context-sensitive critique of the methodological turn toward self-identification in quantitative research.

The paper is structured as follows: Section 2 introduces the concept of self-identification and outlines its use in survey research internationally and in Germany. Section 3 critically assesses the epistemological challenges of self-identification variables, particularly their potential to essentialize and stabilize race. The final and fourth section closes with methodological implications and recommendations.

## 2 Self-identification in quantitative research

The concept of self-identification in surveys has undergone significant evolution, shaped by both internal developments and external pressures. Historically, national censuses documented basic demographic information, such as fertility, mortality, and employment, with little attention paid to racial or ethnic identity (Kukutai and Thompson, 2015). However, civil rights movements in the U.S. during the 20th century catalyzed a shift toward allowing individuals to define their own racial and ethnic identities, rather than having such classifications imposed on them by census takers (Supik, 2023). This turn marked a move toward more subjective and fluid definitions of identity (Simon et al., 2015).

### 2.1 International use of self-identification in surveys

In the past, the practice of self-identification was well-established as a population policy tool before being adopted into social science research. For instance, classifications based on “ethnicity” or “race” were already present in official U.S. censuses dating back to 1790 (Bennett, 2000). Similarly, Canada has implemented self-identification measures in its census, using categories influenced by its multicultural policies (Simon et al., 2015). In Latin America, countries have also adopted self-identification approaches, though with distinct differences in how they uniquely define and measure race and ethnicity (Cabella and Porzecanski, 2015, p. 175). In some cases, self-identification data has been used to protect minorities and promote equality, while in others, it has been employed to maintain control over marginalized groups (Kukutai and Thompson, 2015, p. 41). This highlights the ambivalent nature of identification variables, which can serve as both a tool for empowerment and a mechanism for reinforcing social hierarchies.

Despite the widespread adoption of self-identification among surveys, there is significant variation in its implementation across countries and regions. In North America and Oceania, it is common to ascertain ethnic classification, while European countries have been more reluctant to include these in official forms, often due to historical misuse of racial data (Morning, 2008, p. 263). France, Germany, Spain, Belgium, Denmark, and Italy, for example, have excluded ethnic categories from their official statistics due to political, legal, and constitutional concerns aimed at preventing social divisions (Simon et al., 2015, p. 12). The UK is a notable exception. Initially hesitant about collecting race and ethnicity data, the UK has now institutionalized an ethnic monitoring, especially following the passage of the 2010 Equality Act, which holds public institutions accountable to regularly report on equality data (Gillborn et al., 2018, p. 162f.; Supik, 2017, p. 198). The UK's approach, based on its national census, provides a broad range of response options, including 18 different ethnic categories and the ability for individuals to self-identify using their own terms. This flexibility accommodates the fluidity of racial and ethnic identities (Aikins and Supik, 2018). While this model has proven applicable within the UK, it complicates cross-national comparisons, as different countries use varying definitions of identity, with some focusing on cultural affiliation or religion rather than race or ethnicity (Simon et al., 2015, p. 6).

### 2.2 Self-identification in the German context

In Germany, the incorporation of self-identification into surveys is relatively new and remains highly sensitive due to the country's historical abuse of racial data, particularly during the Nazi era. The reluctance to engage with racial and ethnic categories in official statistics has been further compounded by the persistent denial of racism within contemporary German discourse, which often frames racism as a foreign or historical problem (Bojadžijev et al., 2017; Chin et al., 2009; Messerschmidt, 2011). Terms such as “xenophobia” (“Fremdenfeindlichkeit”) have been employed to depict racism primarily as anti-foreigner sentiment, thus obscuring the structural nature of racism in Germany (Mecheril and Scherschel, 2011; Terkessidis, 2018).

However, more recent incidents, such as the attacks in Hanau and Halle, combined with the influence of the global Black Lives Matter movement, have pushed scholars and policymakers in Germany to reconsider how racial and ethnic identities are measured. Today, the use of closed self-identification formats dominates the imprint of many questionnaires (DeZIM, 2023b; Kronenbitter et al., 2023; Salikutluk and Podkowik, 2024). Closed formats, which use predefined categories, are practical for survey completion and help reduce non-responses. These formats play a crucial role in identifying general trends and patterns, especially when large-scale data collection is involved. Nonetheless, this methodological strength comes with trade-offs: such simplification may obscure the fluid, context-dependent, and intersectional nature of racial identities. Rather than dismissing closed formats outright, it is crucial to recognize their analytical utility while also exploring complementary approaches—such as hybrid or open-ended questions—that can better capture the complexity of identities without undermining statistical coherence (Roth, 2016).

## 3 Employing self-identification in surveys: theoretical challenges

The shift toward self-identification in surveys represents a significant methodological development, although some questions remain unresolved. Variables in surveys do more than gather data; they actively shape, and are shaped by, social realities. Censuses, for instance, are not just tools—they serve as platforms where concepts like race and ethnicity are negotiated and reified (Simon et al., 2015). Drawing on critical theories of race and racism, two key critiques emerge regarding the use of self-identification: first, the technical limitations associated with distinct racial categories in statistics, and second, the risk of reifying race as a fixed category.

### 3.1 Static categories for dynamic realities

While self-identification provides a practical mechanism to assess racial and ethnic identities, it inherently falls short in capturing the nuanced, evolving nature of personal identity. Individuals do not possess fixed identities; they adapt their sense of self across different contexts throughout their lives (Ludwig, 2019, p. 2748). This rigid categorization creates the illusion that identity is static, while in reality, it is socially constructed and dynamic. According to Wimmer (2008), the belief that ethnic or cultural groups are naturally distinct stems from Herder's ideas of discrete cultures. He then challenges this idea by demonstrating that ethnic boundaries are not inherently given but are socially constructed, politically delineated, and actively maintained (Wimmer, 2008). The use of simplified racial and ethnic categories in surveys therefore risks oversimplifying the complexity of lived identities, which often involve multiple affiliations, transnational connections, and hybrid identifications (Roth, 2016). Brubaker (2002 p. 163) refers to this tendency as “groupism”—the inclination to treat ethnic and racial groups as unitary entities, ignoring their internal diversity in the pursuit of statistical coherence. While such measures might reveal patterns of discrimination, they also risk distorting the complexity of these groups, leading to misrepresentative conclusions that regress outcomes to an independent variable that does not have, strictly speaking, an empirical foundation.

This issue becomes even more challenging when race is treated as an explanatory factor in statistical models. In many cases, quantitative research isolates race as a trait, ignoring the social context in which individuals are treated based on their racial identification (Bonilla-Silva and Zuberi, 2008). Moreover, race, as an independent variable, is often treated as an immutable characteristic in statistical analyses, with other factors—such as class or education—viewed as secondary. This oversimplification contributes to a problem known as the “post-treatment bias” (Sen and Wasow, 2016, p. 500). For instance, incorporating variables like education or income as covariates can lead to biased results, since these variables are not external to race but are structurally co-constituted with it. This creates a major obstacle for quantitative racism research, as it entangles the explanation of racism with its consequences (Sen and Wasow, 2016, p. 504f.). As Hu notes, race is already deeply intertwined with social structures, power dynamics, and historical legacies (Hu, 2019a,b). Hereafter, a “counterfactual causal model” of discrimination, which treats race as a simple treatment effect, ignores the ways in which race is embedded in broader systems of social stratification (Kohler-Hausmann, 2019, p. 1167f.).

As a result, attempts to measure the “direct effects” of race rely on the flawed assumption that race is a static and easily measurable trait. This oversimplifies the complexities of racial experiences by ignoring indirect effects—such as historical segregation, wealth inequality, and systemic discrimination—which are essential for understanding how race operates in society (Reskin, 2012). Feagin and Eckberg's concept of “past-in-present discrimination” emphasizes that historical discrimination continues to shape present-day outcomes examined with quantitative methods (Feagin and Eckberg, 1980). Although many quantitative researchers are aware of the entanglement between race and historical processes, there remains a need to further develop methodologies that can adequately account for these dynamics. At the center of this challenge lies the problem that regression models examining the influence of an independent variable labeled “race” risk misinterpreting the resulting statistical effect as an inherent characteristic of the racialized individual, rather than recognizing it as an outcome of the social forces that produce racial meanings in the first place (Karakayali, 2022).

### 3.2 Risking racial re-inscriptions

Although these critiques on a shortened view on self-identification are widely acknowledged, different scholars propose that the construction of racial and ethnic categories may be necessary for political mobilization. In reference to Spivak's work on the subaltern, the term “strategic essentialism” suggests that marginalized groups might need to temporarily adopt essentialist identities to create a political body that represents group-specific interests (Pande, 2017). Similarly, in his reflections on the concept of ethnicity and cultural belonging, Stuart Hall advocates that while identity is always fluid and in process (Hall, 2012a, p. 26), marginalized communities are often compelled to fix their racial identities to effectively challenge power structures (Hall, 2012b, p. 15). To gain visibility, minorities thus seek to aggregate individual experiences into broader societal patterns to expose systemic inequalities. Without these categories, the diffuse nature of racism may remain hidden, making it harder to hold institutions accountable. Aggregated quantitative data thus becomes a crucial tool to reveal how structural discrimination works within society.

Yet, this strategic essentialism carries risks. As Spivak warned, the prolonged use of these identities may foster “frozen identities and deepen differences over time” (Eide, 2016, p. 2279). In addition, Spivak's original framing of strategic essentialism was situated within class struggle (Spivak, 1987, p. 205). As such, the category of working class could be, in theory, dissolved once economic inequalities were abolished, while for race, the situation turns out to be more complex since race constitutes a “sliding signifier” (Hall, 2017, p. 32) that intersects with related concepts such as culture, religion, and nationalism (Balibar, 1991, p. 22; Gilroy, 1990, p. 75). Boger deepens this analysis with her trilemma concept, which argues that when marginalized groups employ essentialist identities to gain visibility and empowerment, it becomes impossible to simultaneously deconstruct these identities. Efforts to normalize, empower, and deconstruct racial identities involve trade-offs, where achieving two of these goals inevitably excludes the third alternative (Boger, 2019, p. 167). Therefore, and if quantitative measures of self-identification are used for political empowerment, a simple departure from strategic essentialist standpoints becomes impossible, since the struggle for social justice roots in the articulation of specific racialized categories.

Here lies one major challenge that is tied to the use of self-identification—especially when approached through a strategic essentialist lens. Although self-identification is often framed as a flexible, anti-essentialist concept (Aikins and Supik, 2018), it is not free from the influence of broader social dynamics. Identity, including racial identity, is not something that originates solely within the individual. As Hall (2006, p. 186) argues, people come to understand themselves through a social process: they see themselves, in part, through the eyes of others. This means that even when individuals are asked to self-identify—such as by selecting a category on a survey—the options available and the meanings attached to them are already shaped by societal norms. What appears to be a personal choice is therefore embedded in structures that reproduce racial boundaries. Given that racism itself operates through this relational logic, why would the concept of self-identification escape these dynamics? Balibar warned that racist ideologies do not remain static but evolve in response to societal transformations and racial struggle. In what he calls “neo-racism,” explicit racial language is replaced by discourses centered on culture, heritage, or national identity. This culturalist turn should not be seen as a retreat from racism, but as a strategic adaptation, allowing exclusionary practices to persist under the guise of protecting cultural integrity (Balibar, 1991). Neo-racism, therefore, does not revolve around biological differences but rather the fear of cultural pollution and ambiguity (Hall, 2006, p. 186). In this ‘racism without races', traditional racial hierarchies are recast as narratives of cultural incompatibility. When we consider self-identification as a means of describing ethnic groups in Germany, we see that it may inadvertently reinforce these culturalist exclusions. Racism, as a system, seeks to fix the “racialized other” in place and prevent their return to the sphere of normality (Hall, 2006, p. 186), thereby reproducing the very idea of distinguishable races, even if they are not explicitly coded in a biological way (Bojadžijev, 2015; Lentin, 2014).

In their concept of *racecraft*, the Fields sisters make a compelling argument about the ambiguous origins and enduring nature of racial categories. They describe racecraft as a process that “occupies a middle ground between science and superstition, an invisible realm of collective understandings, a half-lit zone of the mind's eye” (Fields and Fields, 2022, p. 23). This idea emphasizes that race does not emerge from a single, identifiable source (i.e., belief in biological differences) but is constructed through a complex web of shared social beliefs and cultural practices. Much like witchcraft, racecraft thrives on collective understandings, even though its basis is not biologically grounded (Fields and Fields, 2022, p. 8). As they further observe, these practices make race “real” by transforming the effects of racism into false causalities. This logic is evident in statements such as “Black southerners were segregated because of their skin color”—a sentence that “makes segregation disappear as the doing of segregationists and then […] reappears as a trait of only one part of the segregated whole” (Fields and Fields, 2022, p. 17). In the same way, self-identification variables risk naturalizing race by presenting it as a given characteristic of individuals, rather than as the outcome of racializing processes—which overlooks that racism, and that is the key understanding of the term racecraft, has already been on the scene (Fields and Fields, 2022, p. 19). For instance, racial identification on administrative forms (e.g., census data or medical records) reinforce the very idea of race being an objective and permanent category (Fields and Fields, 2022, p. 47f.). These societal rituals serve as platforms through which racial categories take on a life of their own: by requiring individuals to identify with specific racial categories, as in quantitative statistics, these practices make race material in ways that affect how individuals see themselves and how institutions treat them.

To understand how institutional practices produce racial realities, a look to Butler might be helpful. According to her theory of performativity, gendered identity is not a “being” but constituted through regulatory procedures of gender coherence (Butler, 1999, p. 33). Just as Butler argues that gender is performed into existence, racial categories too are continuously performed, regardless of one's cautious efforts to put “race” in quotation signs. This relates to Butler's concept of subjectivation, by which (racial) identities are both recognized by and subjected to societal norms (Butler, 2001, p. 18f.). In a similar fashion, Goldberg (1992) emphasizes that racial categories are not merely an objective representation of things but actively form the world they describe (Goldberg, 1992). In other words, no representation simply reflects reality. Instead, it mediates and reconstructs it through its own logic, such as a screen's use of light or a photograph's reliance on the photoelectric effect. Representations involve reductions and selective framing—maps simplify cities, and flags symbolize nations—demonstrating that they are never identical to what they depict but actively reconfigure the things they aim to present.

Surveys that require individuals to categorize themselves by race do not merely reflect social realities—they actively shape them. For instance, Roth (2016) shows how individuals adjust their self-categorization depending on how they expect surveyors to interpret these categories. This suggests that statistical practices not only reflect but may also contribute to the materialization of racial identities in both institutional and everyday contexts. As a result, identification variables, though mostly used as antiracist means, can align with the very logic of racist discourses that eventually emphasize relations of difference and belonging rather than breaking it (Broden and Mecheril, 2010, p. 17).

## 4 Discussion

“Theory is always a detour on the way to something more important”

(Hall, 2012c, p. 66)

While self-identification provides valuable insights into how individuals perceive their racial identities, its uncritical use risks reinforcing race as a fixed, essentialized category rather than deconstructing its socially constructed nature. This observation is especially relevant when examining how theory can unsettle methodological conventions, as this paper has aimed to show. As Hall (2017) describes, race remains a “master concept,” persistently organizing societal relations even when stripped of biological determinism. Categories like “Turkish” or “German,” while appearing as neutral national identities, are deeply entwined with assumptions about cultural difference (Broden and Mecheril, 2010). Moreover, translating these categories into quantitative data often gives them an illusory permanence and objectivity, perpetuating stereotypes and obscuring systemic inequalities (Lentin, 2020). Omi and Winant (1994) emphasize that racism is a social project tied to essentialist categories, making it imperative for researchers to engage with race's relational and dynamic nature. Quantitative approaches must move beyond rigid frameworks, acknowledging the fluidity and intersectionality of identity to avoid unintentionally reinforcing the very structures they aim to critique.

### 4.1 Methodological implications for future research

Critiques of self-identification as a quantitative measure often raise concerns about its potential to reinforce racial distinctions and essentialist thinking. Such critiques might suggest that self-identification should not be used in racism research. However, this paper argues that, despite its limitations, self-identification remains a vital tool for understanding and addressing racism—provided it is applied with careful methodological design and critical reflection. To harness its potential while mitigating its risks, future research methodologies must adopt reflexive, intersectional, and context-sensitive approaches. This involves not only innovative data collection and analysis methods but also a deeper theoretical engagement with the complexities of race and identity. Below, key methodological considerations are drawn to provide a more detailed roadmap for future research:

1. Multiple Identification Variables to measure racialization: Incorporating both self-identification and self-perceived external attribution can illuminate the dynamic interplay between internal identity formation and external racialization. While self-identification captures how individuals position themselves within available identity frameworks—making it particularly useful for analyzing political affiliations, self-understanding, or group-based mobilization—external attribution reflects how individuals are perceived and categorized by others based on socially coded markers such as phenotype, name, language, or cultural cues. Rather than treating these measures as interchangeable, they should be understood as capturing different yet interconnected aspects of racialization. At the same time, using both measures in tandem can expose discrepancies between internal identity and external categorization, thereby shedding light on how racialization is negotiated at the intersection of agency and structure. This dual approach not only enriches empirical insights but also helps theorize racialization as a relational process, rather than a static trait (Aikins and Supik, 2018).
2. Context-Sensitive Categories: Rigid racial categories often fail to capture the fluid and context-dependent nature of identity. Surveys should allow for more flexible, open-ended responses that acknowledge the multiple and overlapping influences shaping identity. Such categories allow for the incorporation of historical, social, and political contexts, recognizing that identity is not static but constantly negotiated. For example, some surveys have experimented with hybrid approaches, such as allowing participants to provide detailed self-descriptions alongside selecting from broader, predefined categories. This approach complements traditional methods by offering richer qualitative insights while maintaining comparability in quantitative analyses. For instance, Roth (2016) examined the use of open-ended racial and ethnic questions in U.S. surveys and demonstrated how such methods can reveal nuanced identity markers that predefined categories often obscure, such as regional or cultural affiliations. Relatedly, Lentin (2014) highlighted cases where integrating narrative-based identity questions with quantitative frameworks provided a deeper understanding of how individuals situate themselves within systems of racialization. These examples suggest that flexible categories can lead to more accurate representations of identity, particularly in societies where racial and ethnic classifications are highly contested or fluid. However, these methods also present challenges. Open-ended responses can complicate data coding and analysis, making it harder to compare results across studies or populations. Additionally, they may introduce biases if researchers interpret responses through their own cultural or theoretical frameworks.
3. Intersectional Frameworks: Incorporating intersectionality into quantitative research is a significant challenge that has sparked considerable debate among scholars. Intersectionality, as conceptualized by Crenshaw (1989), highlights how overlapping forms of discrimination and privilege—such as race, gender, class, and migration status—interact to produce unique experiences of marginalization. In practice, survey researchers can incorporate intersectionality by (a) designing surveys that explicitly ask respondents to describe overlapping experiences of marginalization (e.g., being a racialized woman in a particular institutional context), (b) creating interaction terms in statistical models that capture compound identity positions—while also clearly stating their interpretive limits, and (c) piloting items that explore intersectional dynamics qualitatively before translating them into survey form. For instance, cognitive pretests might uncover how participants understand intersectional categories before they are scaled up in large datasets.
4. Qualitative Methods to Complement Quantitative Data: While quantitative surveys can reveal broad patterns and disparities, they often obscure the complexities and contradictions inherent in lived experiences of race and identity. Complementing quantitative data with qualitative methods such as interviews, ethnographies, or focus groups allows researchers to explore the nuances of identity construction and racialization. To effectively integrate qualitative insights into survey research, mixed-methods designs should be structured sequentially or simultaneously. For example, qualitative pre-studies can identify context-specific identity terms that inform survey categories. Alternatively, follow-up interviews with select survey participants can deepen the understanding of why individuals choose certain identity labels, and in which contexts.
5. Critical Reflection on Variable Construction: The construction of racial variables requires careful consideration to avoid reifying race as a static and universal category. Researchers must engage critically with the process of operationalizing race, recognizing that racial classifications are not natural but socially and historically contingent. For example, the category “Black” in the United States carries different connotations and histories than in Brazil or South Africa. This variability highlights the importance of designing racial variables that are sensitive to local and global contexts while remaining grounded in critical theoretical perspectives on race (Hall, 2017; Ludwig, 2019).
6. Reframing Causal Language and Models: Race is often treated as an independent variable in causal models, which can obscure its social construction and relational nature. Researchers must instead conceptualize race as a product of broader structural, historical, and political processes. This involves shifting from simplistic causal explanations to relational models that examine how race interacts with other variables, such as socioeconomic status or institutional discrimination (Hu, 2020; Sen and Wasow, 2016). By reframing causal language, researchers can avoid implying that racial disparities are inherent to racial groups and instead emphasize the systemic factors driving such disparities.
7. Methodological reflexivity: Reflexivity is crucial for ensuring that research methodologies do not inadvertently reinforce the very racial categories they seek to deconstruct. This requires researchers to critically examine the assumptions underlying their methods, and the broader implications of their work. For instance, how does the framing of survey questions reflect dominant cultural narratives about race? Are the categories used in research reproducing essentialist understandings of race? Engaging in ongoing reflexive practices helps researchers navigate these challenges and align their methods with critical theories of race and racism (Brubaker, 2002; Fields and Fields, 2022).
8. Ethical Considerations in Data Representation: Beyond methodological reflexivity, researchers must also consider how racial data is represented and interpreted. For instance, when disparities between racial groups are highlighted, care must be taken to avoid framing these differences as inherent to the groups themselves (Wimmer, 2008). Instead, disparities should be contextualized as products of structural inequalities.

## 5 Conclusion

This paper critically examined the role of self-identification in quantitative surveys, exploring both its potential to capture the lived realities of racialized individuals and its risks of reifying race as a fixed, measurable category. Drawing on critical race theory and sociological perspectives, the analysis underscores that self-identification—while valuable as a methodological innovation—can also reproduce essentialist understandings of identity when embedded in survey instruments without adequate theoretical and contextual reflection. A key objective of this paper has been to stress that quantitative approaches to racism research are not inherently flawed. On the contrary, they have made significant contributions to documenting structural inequalities and informing anti-discrimination policy. Recent adaptations in U.S. federal surveys illustrate how racial data collection can evolve in reaction to these very concerns. In 2024, the Office of Management and Budget (OMB) revised its statistical standards for race and ethnicity data for the first time since 1997. These changes aimed to reduce the reification of racial categories by introducing more flexible and inclusive practices. Among the most significant updates is the adoption of a combined race and ethnicity question, replacing the traditional two-question format. This change acknowledges that racial and ethnic identities are often intertwined and resists the artificial separation of categories. Perhaps most notable is the OMB's explicit commitment to periodically reviewing these standards—an institutional recognition that racial categories are socially constructed, historically contingent, and in need of continuous reevaluation (United States Census Bureau, 2024).

Yet, the construction of identification variables requires careful navigation—particularly in contexts like Germany, where historical legacies of racial categorization, combined with institutional reluctance to address race directly, shape both the possibilities and limitations of data collection. As Germany begins to incorporate race-sensitive variables into large-scale surveys, the challenge is to do so in ways that do not inadvertently stabilize the very categories they aim to make visible. Theoretical and methodological considerations such as reflexivity, hybrid measurement strategies, and sensitivity to national context are therefore not abstract concerns but urgently needed tools for shaping a data infrastructure that supports anti-racist knowledge production. Self-identification, in this light, is neither inherently progressive nor problematic–it captures exactly what it is designed to capture: individuals who tick a predefined box in a questionnaire. But whether these individuals are indeed the most affected by racism—or whether others remain invisible—cannot be answered by the measure itself. This is precisely why, as Hu notes, racism cannot be easily isolated within quantitative research designs. Rather, it materializes through social relationships and must be understood as a dynamic process. Even categories that seem neutral—such as those referencing nationality, culture, or ethnicity—can become entangled with racial meaning, particularly when they are used to sort people into groups assumed to differ in mentality, values, or behavior (Broden and Mecheril, 2010, p. 14f.). Such classifications risk reinforcing hierarchies that are structurally and discursively produced. As Mecheril and Broden argue, the ambiguity of ethnic or national categories is not accidental; it is precisely this ambiguity that allows racist logics to persist under seemingly benign labels (Mecheril, 2014, p. 15).

These insights remind us that identification categories—whether racial, ethnic, or national—never operate outside of the broader social and historical forces that shape them. As Balibar (1992) has shown, racism can take on new forms through humanist or culturalist discourses, making it difficult to identify and resist. Even when biological definitions of race are formally rejected, they often “sidle back through the pantry window,” as Hall warns (Hall, 2017, p. 37).

Thus, rather than discarding categories like race, the task is to work with them critically—to question their construction, their function, and their consequences. This demands a methodological orientation grounded in continuous critique and reconfiguration. Hall's observation that “theory is always a detour on the way to something more important” (Hall, 2012c, p. 66) is particularly relevant here. Theory, when brought into dialogue with quantitative practice, can help prevent race from regaining authority—even, and especially, when we think that we have already moved beyond it.

## Data Availability

The original contributions presented in the study are included in the article/supplementary material, further inquiries can be directed to the corresponding author.
